# The lncRNA TEX41 is upregulated in pediatric B-Cells Acute Lymphoblastic Leukemia and it is necessary for leukemic cell growth

**DOI:** 10.1186/s40364-021-00307-7

**Published:** 2021-07-07

**Authors:** Francesca Maria Orlandella, Giovanni Smaldone, Giuliana Salvatore, Luigi Vitagliano, Alessandra Cianflone, Rosanna Parasole, Giuliana Beneduce, Giuseppe Menna, Marco Salvatore, Peppino Mirabelli

**Affiliations:** 1 IRCCS, SDN, Via E. Gianturco 113, 80143 Naples, Italy; 2 Dipartimento di Scienze Motorie e del Benessere, University of Naples Parthenope, Via Medina 40, 80133 Naples, Italy; 3 CEINGE - Biotecnologie Avanzate S.c.a.r.l, Via Gaetano Salvatore 486, 80145 Naples, Italy; 4 Institute of Biostructures and Bioimaging, C.N.R, Via Mezzocannone 16, 80134 Napoli, Italy; 5 Department of Pediatric Hemato-Oncology, AORN Santobono-Pausilipon, Naples, Italy

**Keywords:** lncRNA, B-Cells Acute Lymphoblastic Leukemia (B-ALL), Biomarker, Diagnosis

## Abstract

**Background:**

Long non-coding RNAs (lncRNAs) represent a diverse class of RNAs involved in the regulation of various physiological and pathological cellular processes, including transcription, intracellular trafficking, and chromosome remodeling. LncRNAs deregulation was linked to the development and progression of various cancer types, such as acute leukemias. In this context, lncRNAs were also evaluated as a novel class of biomarkers for cancer diagnosis and prognosis. Here, we analyzed TEX41 in childhood B cell acute lymphoid leukemia (B-ALL).

**Methods:**

Total RNA was extracted from pediatric B-ALL patients (at diagnosis and after induction of therapy) and from healthy subjects. Total RNA was also extracted from different leukemia cell line models. The expression level of TEX41 was evaluated by q-RT-PCR. Also, the dataset deposited by St. Jude Children’s Research Hospital was consulted. Furthermore, the silencing of TEX41 in RS4;11 cell line was obtained by 2′-Deoxy, 2′Fluroarabino Nucleic Acids (2′F-ANAs) Oligonucleotides, and the effect on cell proliferation was evaluated. Cell cycle progression and its regulators were analyzed by flow cytometry and immunoblotting.

**Results:**

We exploited the St Jude Cloud database and found that TEX41 is a lncRNA primarily expressed in the case of B-ALL (*n* = 79) while its expression levels are low/absent for T-cell ALL (*n* = 25) and acute myeloid leukemia (*n* = 38). The association of TEX41 with B-ALL was confirmed by real-time PCR assays. TEX41 disclosed increased expression levels in bone marrow from patients with B-ALL at diagnosis, while its expression levels became low or absent when retested in Bone Marrow cells of the same patient after 1 month of induction therapy. Also, silencing experiments performed on RS4;11 cells showed that TEX41 downregulation impaired *in vitro* leukemic cell growth determining their arrest in the G2-M phase and the deregulation of cell cycle proteins.

**Conclusions:**

Our findings highlight that TEX41 is an upregulated lncRNA in the case of B-ALL and this feature makes it a novel potential biomarker for the diagnosis of this leukemia subtype in pediatric patients. Finally, TEX41 expression seems to be critical for leukemic proliferation, indeed, silencing experiments targeting TEX41 mRNA in the RS4;11 cell line hampered *in vitro* cell growth and cell cycle progression, by inducing G2-M arrest as confirmed propidium iodide staining and by the upregulation of p53 and p21 proteins.

**Supplementary Information:**

The online version contains supplementary material available at 10.1186/s40364-021-00307-7.

## Background

Acute leukemias originate from the malignant transformation of myeloid or lymphoid hematopoietic progenitors normally residing in bone marrow (BM). Due to their molecular and cellular alterations, the leukemic cells can evade the physiological mechanisms controlling terminal differentiation and cellular proliferation. In this way, actively proliferating leukemic blasts invade the bone marrow and prevent the growth of normal blood cells [1]. The most common type of blood cancer in children is represented by Acute Lymphoblastic Leukemia (ALL) occurring in more than three-quarters of all childhood leukemias [2, 3]. Generally, about 80 % of childhood ALL are classified as B cell type, and despite the progress in the cure, this cancer type remains the major cause of death in children worldwide. The identification of new biomarkers and the understanding of the complex molecular mechanism of the disease is still considered challenging for the scientific community [4]. In this context, long non-coding RNAs (lncRNA) are emerging as a new class of molecules involved in the molecular alterations leading to the onset of ALL. Specifically, lncRNAs are defined as a non-coding-protein RNA longer than 200 nucleotides. After transcription by RNA Polymerase type II and post-transcriptional modifications, a lncRNA can regulate the gene expression at multiple levels in physiological as well as pathological conditions [5, 6]. Recently, the importance of lncRNA is highlighted by their involvement in a wide range of cellular function based on subcellular localization: in the nucleus, lncRNAs can regulate the chromatin organization and control the transcription activity, while in the cytoplasm lncRNAs are involved in the stability of mRNA and in the regulation of translation [7]. This spectrum of functions is possible since lncRNAs can act at multiple levels, through their interactions with microRNAs or by binding to several regulatory proteins such as transcription factors, epigenetic enzymes, or nuclear hormone receptors. For these reasons, lncRNAs are involved in the various biological processes contributing to the regulation of cell proliferation, differentiation, angiogenesis, apoptosis, and cell motility [8].

Recent advances in oncology research unveiled the aberrant expression of lncRNAs in many human diseases, especially during carcinogenesis where they may act as potential tumor-suppressor or oncogene[9–11].

Moreover, lncRNAs can be exploited also as potential diagnostic and prognostic biomarkers in cancer patients since they are easily detectable in body fluids and present a stable expression during cellular differentiation [12]. One of the reasons for the great potential of circulating lncRNAs as biomarkers in clinical practice is due to their resistance to the ribonuclease degradation activities when included in exosomes or apoptotic bodies and, consequently, to more stability. The second reason is that the circulating cancer-associated lncRNAs are detected in blood, plasma, urine, or saliva with noninvasiveness, but high sensitivity and specificity, methods [12, 13].

In this scenario, accumulating pieces of evidence highlighted the relationship between the dysregulation of lncRNAs and leukemia initiation and outcome prediction[14–16].

The function and expression level of several lncRNAs has been investigated in different subtypes of B cell acute lymphoblastic leukemia [17, 18]. Recently, microarray studies allowed to identify the aberrant expression of specific lncRNAs in pediatric B-ALL [19–21]; however, the research conducted so far is inconclusive and further studies are needed.

Here, we focused on the potential role of TEX41, a lncRNA recently identified by our group, as a possible new biomarker in pediatric B-ALL [22].

We found that TEX41 is significantly over-expressed in pediatric B-ALL patients compared with healthy subjects. Moreover, the expression level of TEX41 decreases after chemotherapy suggesting that this lncRNA is a potential marker of disease onset and of the progression of the pathology.

## Materials and methods

### Study population

The study population was composed of ten young patients affected by common B-ALL consecutively admitted to A.O.R.N. Santobono-Pausilipon Hospital of Naples (Italy). At diagnosis, a bone marrow aspiration was performed to evaluate disease characteristics such as blasts morphology, immunophenotype, cytogenetic and molecular abnormalities as reported in Table 1.

**Table 1:** Clinical features of B-ALL pediatric patients enrolled for the analysis of TEX41 expression

PATIENT ID	Pt 1	Pt 2	Pt 3	Pt 4	Pt 5	Pt 6	Pt 7	Pt 8	Pt 9	Pt 10
**Age (years)**	3,6	4,5	2,9	11,1	10,9	15	10,2	9,9	14,8	4.9
**Sex**	M	M	F	M	F	F	M	F	M	M
**Race**	Caucasian	Caucasian	Caucasian	Caucasian	Caucasian	Caucasian	Indian	Caucasian	Caucasian	Caucasian
**WBC**	67,550/mmc	n.d.	n.d.	3790/mmc	4730/mmc			120,690/mmc	453,600/mmc	
**BM blast(%)**	68	80	70	65	89	50	80	90	95	70
**Immuno-phenotype**	B common	B common	B common	B common	B common	B common	B common	B common	B common	B common
**Molecular biology**	t (1;19) E2A/PBX1 rearrangement	t (12;21) TEL/AML1 rearrangement	Negative	Negative	Negative	Negative	Negative	Negative	Negative	t (12;21) TEL/AML1 rearrangement
**Cytogenetic**	45, XY, i (9)(q10), -13, add (19)(p13)[8]/46, XY,[8]	46,XY	46,XX	59, XXXY, + 4,+4,+5,+6, + 10 + 14,+17,18,+21,+21[5]/46, XY, [10]	NE	46, XX	NE	NE	46, XY	46, XY
**GPR day + 8**	Yes	Yes	Yes	Yes	Yes	Yes	Yes	No	Yes	Yes
**BM blast < 10 % day + 15**	Yes	Yes	Yes	Yes	Yes	Yes	Yes	No	Yes	Yes
**NCI risk**	High	NE	High	High	High	High	High	High	High	High
**MRD risk**	Standard	Intermediate	Intermediate	Standard	Intermediate	NE	NE	NE	NE	NE
**Tex41**	0,00048571	0,00003693	0,00004515	0,00120526	0,00001881	0,00003052	0,00001824	0,00001256	0,00001052	0,00107202

*WBC* white blood count; *BM* bone marrow blast; *GPR* good prednisone responder; *NCI* national cancer institute; *MRD* minimal residual disease; *NE* not evaluable (absence of metaphases)

The present study has been approved by the local ethical committee (Comitato Etico IRCCS Pascale, Naples Italy) of IRCCS-SDN with protocol number 6/16 of the 14/09/2016 and by the local ethical committee of AORN Santobono-Pausilipon (Comitato Etico Cardarelli/Pausilion, Naples Italy) with number 94 of 08/02/2017 following relevant guidelines and regulations. All participants provided informed consent signed by parents.

All samples used in this study were processed and stored at IRCCS SDN Biobank that is a member of the Italian node of the BBMRI-ERIC infrastructure [23].

### B-ALL patients immunophenotype

For detection of blasts in Bone Marrow Blood samples derived from B-ALL patients, we used the Cytoflex V2-B4-R2 (Beckman-Coulter, Brea, CA, USA) after staining cells with CD45FITC-CD56PE-CD19ECD-CD3PC5 antibody mix (Beckman Coulter #6,607,073). Briefly, 100 µL of Bone marrow Blood was labeled with 5 µL of antibody mix for 30 min at RT. Then cells were washed with Phosphate Buffer Saline (PBS) at pH 7.4 and analyzed with Cytoflex. Data were analyzed with Kaluza Analysis Software 2.1 (Beckman Coulter).

### St. Jude Children’s Research Hospital database analyses

We use three different databases recovered from St. Jude Children’s Research Hospital (https://platform.stjude.cloud/data/cohorts/pediatric-cancer). We analyze the Feature Counts of TEX41 in 3 different databases: Pan-Acute Lymphoblastic Leukemia (PanALL, SJC-DS-1009) that contains RNAseq data derived from 79 pediatric B-ALL patients; Acute Myeloid Leukemia dataset that contains RNAseq data derived from 38 AML patients (the patient ages are not disposable); T-cell Acute Lymphoblastic Leukemia dataset that contains RNAseq data derived from 25 pediatric T-ALL patients.

### BM mononuclear cells isolation

Bone marrow mononuclear cells were purified according to density gradient purification protocol performed at the SDN Biobank. Briefly, BM blood was diluted 1:5 with DPBS w/o Ca2 + and Mg2+ (Gibco) and then stratified on Pancoll (1.077 gr/mL; PAN-Biotech GmbH Aidenbach, Germany) density gradient for centrifugation at 400xg for 30 min. Then, after two wash steps in DPBS enriched with 2 % FBS (Gibco), about 5 × 106 cells were diluted in Trizol reagent for RNA purification and the remaining part was distributed in aliquots (5 × 106 cells/mL) and cryopreserved in liquid nitrogen. In the case of BM from B-ALL cases, the isolated mononuclear cell population was composed of about 80–90 % of lymphoid blasts. BM samples from patients after chemotherapy produced low cell numbers (about 1 × 10^6^ total cells) after density gradient centrifugation and were diluted in Trizol for RNA extraction protocol.

### Cell cultures

Acute lymphoblastic leukemia cell lines were validated for Short-Tandem Repeat profiling (STR) at Deutsche Sammlung von Mikroorganismen und Zellkulturen (DSMZ, Braunschweig, Germany) in agreement with international good laboratory practice [24]. Cells were cultured in Iscove’s Modified Dulbecco’s Media (IMDM) (Thermo Fischer Scientific) supplemented with 10 % of Fetal Bovine Serum (FBS, Thermo Fischer Scientific) and 1 % Glutamax (Invitrogen). All cultures were incubated at 37 °C and 5 % CO2 and seeded in 24 well plates.

### RNA extraction and q-RT-PCR

Total RNA was extracted from cultured cells, from B-ALL patients (at diagnosis and follow up) and from healthy subjects by using Trizol Reagent protocol (Thermo Fischer Scientific, Ma, USA). After extraction, RNA was quantified with Qubit 4 Fluorometer (Thermo Fischer Scientific).

Next, 1 µg of total RNA from each sample was reverted in cDNA using SuperScript III First-Strand Synthesis SuperMix kit (Thermo Fisher Scientific) according to the manufacturer’s protocol.

The expression level of TEX41 was measured by q-RT-PCR using the following formula: 2^−∆Ct^ on C1000 Touch Thermal Cycler (Bio-Rad, CA, USA) using iQ SYBR Green Supermix (#1,708,882, Bio-Rad). Ribosomal Protein S18 (RPS18) level was used as an endogenous control to normalize TEX41 expression.

The following forward (fw) and reverse (rev) primers were used:

RPS18:

fw 5’-CGATGGGCGGCGGAAAATA-3’;

rev 5’-CTGCTTTCCTCAACACCACA-3’.

TEX41:

fw 5’-TCATCTGTGAGGACCGTGAC-3’;

rev 5’-AGCACAGGAGAAGCTGAGTT-3’.

CCNA1:

fw 5’- AAATGGGCAGTACAGGAGGA-3’;

rev 5’-CCACAGTCAGGGAGTGCTTT-3’.

CCNB1:

fw 5’-CATGGTGCACTTTCCTCCTT-3’;

rev 5’ AGGTAATGTTGTAGAGTTGGTGTCC-3’.

CCNE1:

fw 5’-GGCCAAAATCGACAGGAC-3’;

rev 5’-GGGTCTGCACAGACTGCAT-3’.

CCND1:

fw 5’-GCTGTGCATCTACACCGACA-3’;

rev 5’-TTGAGCTTGTTCACCAGGAG-3’.

### TEX41 silencing by 2′-Deoxy, 2′Fluroarabino Nucleic Acids (2′F-ANAs) Oligonucleotides

The silencing of TEX41 in RS4;11 cells was obtained by transfection with 2′-deoxy-2′-fluoro-beta-D-arabinonucleic acid (2′F-ANA) modified oligonucleotides (ASOs) purchased from AUM Biotech (Philadelphia, PA, USA). The sequence of 2’F-ANA against TEX41 is :

5’- AATCAATTCTACCACTATAGC- 3’.

Briefly, 5 × 10^5^ RS4;11 cells were plated in complete media in 24-well plate and 2 µM of 2′F-ANAs against TEX41 and a Scramble 2’F-ANA were tested at 24, 48, and 72 h. After F-ANA treatments, the efficiency of silencing was analyzed by q-RT-PCR.

### Cell proliferation assay

For cell proliferation curves, RS4;11 cell line was plated at the concentration of 1 × 10^6^ cells/well in 6-well plates. After 24, 48, and 72 h of incubation with 2′F-ANA-TEX41 at a concentration of 2 µM, RS4;11 cells were collected and counted at Cytoflex flow cytometer (Beckman Coulter, Germany).

### Flow cytometer analysis

Cell cycle progression was analyzed in RS4;11 cell line after 72 h of incubation with 2 µM of 2′F-ANA-TEX41 and controlled by flow cytometry (FCM) analysis. To this aim, cells were fixed and stained with propidium iodide staining (Beckman Coulter) and a minimum of 10.000 single-cell events were recorded using the CXP software (Beckman Coulter). Then, G0/G1, S, and G2-M phases were determined using Kaluza Analysis Software 2.1 (Beckman Coulter) with the Michael Fox algorithm.

### Western blot

To analyze the expression of cell cycle regulators, immunoblotting was carried out following standard procedures. Briefly, RS4;11 cells transfected for 72 h with 2′F-ANAs against TEX41 or control were lysed and the protein concentration was evaluated by Bradford assay (Bio-Rad, USA). Then, 50 µg of protein extracts were subjected to sodium dodecyl sulfate-polyacrylamide gel electrophoresis (SDS-PAGE), and the nitrocellulose membranes were probed with the following antibodies: anti-CDK4 (#12,790), anti-CDK6 (DCS83), anti-p27/KIP1 (D69C12), anti-p21 (#2947) purchased from Cell Signaling; anti-p-53 (#sc-126, Santa Cruz Biotechnology, Dallas, USA) and anti-α-TUBULIN (#T8203, Sigma-Aldrich). Afterward, the secondary antibodies were used for 1 h at room temperature. After washing, the immune complexes were acquired using the ChemiDoc imaging system (Bio-Rad) coupled with Image Lab software.

### Statistical methods

All results were presented as mean ± SD and the statistical significance was determined with unpaired Student’s t-test using GraphPad 6.0 Software (GraphPad, San Diego, CA). To analyze the expression of TEX41 in the data set of patients, the Mann-Whitney U test, Wilcoxon matched-pairs test, or One-way ANOVA Tukey’s Multiple Comparisons Test were used. The *p-value* < 0.05 was considered statistically significant.

## Results

### Expression of TEX41 in pediatric B-ALL patients samples

**Fig. 1:**
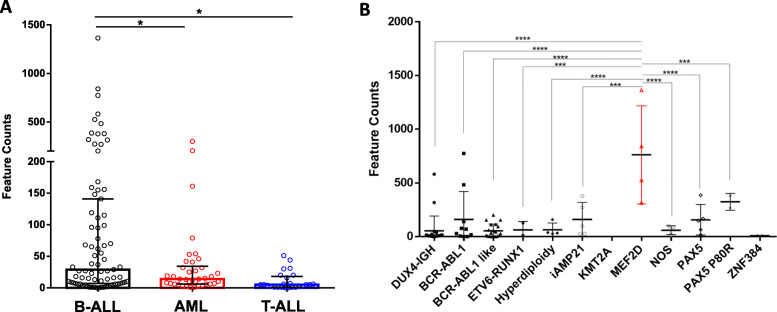
lncRNA TEX41 in St. Jude Children’s Research Hospital databases. **(A)** Feature counts of TEX41 were determined in 79 B-ALL (black circles), 38 AML (red circles), and 25 T-ALL (blue circles) pediatric patients. **(B)** Feature counts of TEX41 were determined in B-ALL patients harboring different chromosomal rearrangement. One-way ANOVA Tukey’s Multiple Comparisons Test was used where *= *p* < 0.05; ***=*p* < 0.001; ****=*p* < 0.0001. Data report the TEX41 feature counts median ± interquartile range.

Recent observations by our research group reported the lnc-TEX41 as one of the most deregulated non-coding genes in pediatric B-ALL patients when compared to peripheral blood B cells from healthy controls [21, 22] (Bio project cod. PRJNA601326). Starting from this preliminary finding, we decided to further evaluate the involvement of TEX41 in leukemogenesis. To this aim, we questioned the St. Jude Children’s Research Hospital cloud database to dispose of a large cohort of RNAseq data on different subtypes of pediatric leukemias (https://www.stjude.cloud/; St. Jude Cloud—a Pediatric Cancer Genomic Data Sharing Ecosystem by Clay MacLeod et al. 10.1101/2020.08.24.264614). Specifically, we used data from 79 B-ALL, 38 Acute Myeloid Leukemia (AML), and 25 T-ALL patients with a mean age of ≈ 15.98, 9.0, and 12.4 years respectively. As reported in Fig. 1 (panel A), TEX41 expression is significantly higher in B-ALL cases when compared to AML (*p* < 0.05, ANOVA Tukey’s Multiple Comparisons Test) and T-ALL (*p* < 0.0001, ANOVA Tukey’s Multiple Comparisons Test). Specifically, as reported in Table 2, the TEX41 median expression in B-ALL is double than in AML (29 vs. 14 reads count) and more than five times in T-ALL (29 vs. 5 reads counts). These results suggest that the lncRNA TEX41 is predominantly expressed by B-ALL leukemic cells instead of AML or T-ALL counterparts.

**Table 2:** Descriptive statistics of data derived from St. Jude Children’s Research Hospital databases

	B-ALL	AML	T-ALL
Number of values	79	38	25
Mean age at diagnosis (years)	15.8	8.9	12.4
Minimum	0.0	1.000	0.0
25 % Percentile	7.000	6.000	1.000
Median	29.00	14.00	5.000
75 % Percentile	141.0	34.25	18.00
Maximum	1364	301.0	51.00
Mean	123.5	35.08	10.72
Std. Deviation	223.5	60.50	14.21
Std. Error of Mean	25.14	9.815	2.842
Lower 95 % CI	73.41	15.19	4.855
Upper 95 % CI	173.5	54.97	16.58
Mean ranks	7.000	6.000	1.000

Furthermore, as reported in Fig. 1 (Panel B) we found that the higher expression levels of TEX41 occurred in patients harboring the MEF2D rearrangement suggesting that the aberrant expression of lncRNA TEX41 could be associated with the epigenetic modifications associated with this chromosomal aberration.

### lncRNA TEX41 expression in B-ALL patients and after chemotherapy treatment

**Fig. 2:**
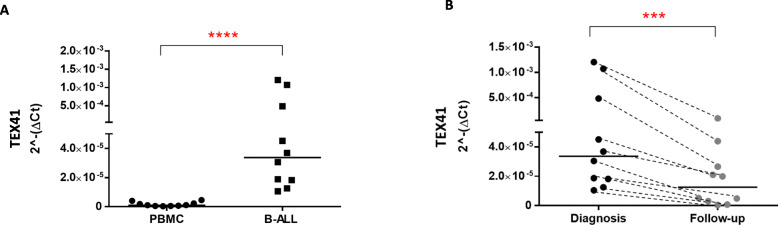
Expression level of lncRNA TEX41 in PBMC derived from healthy subjects and leukemic cells derived from pediatric B-ALL patients. lncRNA TEX41 levels were plotted according to the relative expression (2-ΔCt method) measured in BM cells from patients at diagnosis (B-ALL, *n* = 10, squares) and in PBMC from healthy donors (*n* = 10, circles). ****p < 0.0001, Mann Whitney t-test. **B)** lncRNA TEX41 levels were plotted according to the relative expression (2-ΔCt method) measured in BM cells from patients at diagnosis (*n* = 10, black circles) and after the first chemotherapy cycle (Day + 33, *n* = 10, gray circles). ***p < 0.001, Wilcoxon matched-pairs t-test.

To extend these suggestive *in silico* analyses, we determined the expression level of lncRNA TEX41 in a cohort of 10 pediatric B-ALL patients (Table 1 shows the clinicopathological features of all included cases) in comparison with Peripheral Blood Mononuclear Cells (PBMC) derived from 10 healthy subjects by q-RT-PCR analyses. As shown in Fig. 2 (panel A) the expression levels of TEX41 were significantly higher in B-ALL patients in comparison to PBMCs from healthy subjects, being the median expression level of 3.373*10^− 5^ at diagnosis to 1.033*10^− 6^ in healthy donors (Table 3).

**Table 3:** Statistical parameters for TEX41 expression of B-ALL patients compared to PBMC from healthy donors

*N* = 10	PBMC	B-ALL
**Minimum**	2.950e-007	1.052e-005
**25 % Percentile**	4.788e-007	1.682e-005
**Median**	1.033e-006	3.373e-005
**75 % Percentile**	2.731e-006	0.0006323
**Maximum**	4.565e-006	0.001205

To confirm if TEX41 expression could be associated with B-ALL onset, we tested by q-RT-PCR its expression values in 10 B-ALL patients at the moment of diagnosis and at day + 33 after induction therapy, when the minimal residual disease was undetectable according to the clinical reports (data not shown). As described in Fig. 2 (panel B), the expression of TEX41 significantly decreased (p < 0.01, Wilcoxon) from a median value of 3.373*10^− 5^ at diagnosis to 1.257*10^− 5^ at day + 33 (data summary shown in Table 4). These findings prompted us to evaluate if this lncRNA is important for survival and/or for the proliferation of leukemic B cells as further described.

**Table 4:** Statistical parameters for TEX41 expression of B-ALL patients at diagnosis and after 33 days of therapy

*N* = 10	Diagnosis	Day + 33
**Minimum**	1.052e-005	5.338e-007
**25 % Percentile**	1.682e-005	2.488e-006
**Median**	3.373e-005	1.257e-005
**75 % Percentile**	0.0006323	3.100e-005
**Maximum**	0.001205	9.325e-005

### lncRNA TEX41 expression in human leukemia cell line models and silencing effects in RS4;11 cells

**Fig. 3:**
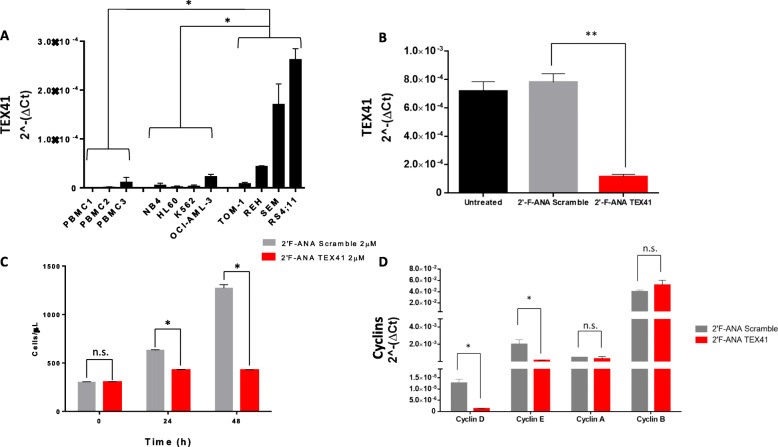
Effect of TEX41 silencing on leukemic cell growth.** (A)** lncRNA TEX41 transcription levels in four B-ALL *in vitro* model systems (TOM-1, REH, SEM and RS4;11), AML *in vitro* model systems (NB4, HL-60, K562, OCI-AML-3), and PBMCs derived from 3 healthy subjects. The relative expression was determined using the 2^−ΔCt^ method. TEX41 relative expression is shown as mean +/− SD of two technical independent experiments. **(B)** Bar-plot showing downregulation of TEX41 levels in RS4;11 cells treated with the 2′F-ANA oligonucleotides designed against lncRNA TEX41 and with 2′F-ANA Scramble for 48 h. The relative expression was determined using the 2^−ΔCt^ method and it is shown as mean +/− SD of two technical independent experiments. **(C)** RS4;11 2′F-ANA Scramble (grey bars) and 2′F-ANA-TEX41 (red bars) treated cells growth (expressed as cells/µL). Cells concentrations are shown as mean +/− SD of three technical independent experiments. **(D)** Cyclins mRNA expression levels in RS4;11 2′F-ANA Scramble (grey bars) and RS4;11 2′F-ANA-TEX41 (red bar) treated cells. The relative expression was determined using the 2^−ΔCt^ method. Cyclins relative expression is shown as mean +/− SD of two technical independent experiments. * = *p*-value < 0.05, ** = *p* < 0.01, Mann Whitney t-test.

Since TEX41 levels in T-ALL patients were low or absent, we decided to test its expression in different continuous B-ALL and AML cell lines to set up subsequent functional studies. Figure 3 (panel A) shows TEX41 expression level in 4 different Acute Myeloid Leukemia (AML; NB4, HL-60, K562 and OCI-AML-3), and Acute Lymphoblastic Leukemia cell lines (TOM-1, REH, SEM, and RS4;11) as well as in Peripheral Blood Mononuclear Cells (PBMC) derived from 3 healthy subjects. The TEX41 expression within the lymphoid group was significantly higher when compared to the myeloid counterpart or normal PBMCs. These results are in agreement with the increased expression of TEX41 in B-ALL than AML blasts or PBMCs. Since the RS4;11 cells disclosed the highest TEX41 expression level when compared to the other cell lines, we selected this model system to silence TEX41 using 2’-F’ANA-ASO antisense nucleotides. As shown in Fig. 3 (Panel B) a 2’-F-ANA against TEX41 was able to significantly reduce its expression after 48 h of incubation. At the same time, Fig. 3 (panel C) shows that RS4;11 cells deprived of TEX41 reduced their *in vitro* cell growth with respect to the counterpart treated with a 2’-F-ANA Scramble. Accordingly, we evaluated the mRNA levels of cyclins involved in the passage from one cycle phase to another. Indeed, Fig. 3 (panel D) disclosed that Cyclins D and E were significantly reduced compared to the 2’F-ANA Scramble treated cells while Cyclins A and B were unchanged. This observation suggests that TEX41 silencing in RS4;11 cells could impair the cell cycle progression inducing growth arrest.

### Silencing effects of TEX41 on cell cycle phase regulation in RS4;11 cells

**Fig. 4:**
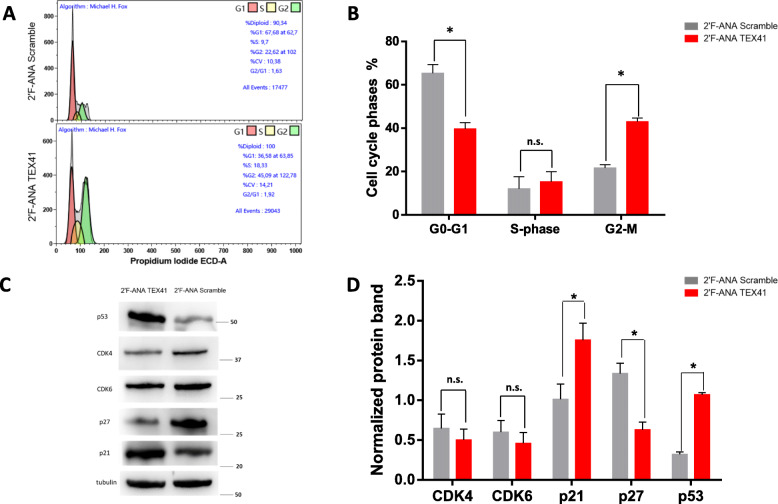
Effect of TEX41 silencing on cell cycle arrest.** (A)** Flow cytometry analysis of cell cycle distribution phases in 2′F-ANA Scramble (upper panel) and RS4;11 2′F-ANA-TEX41 (lower panel) RS4;11 treated cells for 72 h. The image is representative of three independent experiments with similar results. **(B)** The percentage of the cells in the G0-G1, S, and G2-M phase was reported as the mean of three independent experiments +/− SD. Western blot analyses of the indicated protein **(C)** and protein band quantifications **(D)** were performed in RS4;11 2′F-ANA Scramble and RS4;11 2′F-ANA-TEX41 treated cells for 72 h. Numbers represent molecular weight proteins expressed in kDa. ns = not significant; * = *p*-value < 0.05.

To better understand the role that TEX41 could play in blocking cell growth, we performed the FCM analysis of the cell cycle. To enhance the effects of TEX41 silencing, RS4;11 cells were treated for 72 h with 2’F-ANA against TEX41 or Scrambled sequence. At this time point, we found a marked silencing of TEX41 accompanied by the decreased number of cells in comparison with Scramble treated RS4;11 (Supplementary Figure 1). After these treatments, all cells were fixed and stained with propidium iodide to determine the phases of the cell cycle. As shown in Fig. 4 A-B, the silencing of TEX41 in RS4;11 cells induced a significant reduction of the G1 phase and an increase of G2-M when compared to the Scramble-treated cells. The anti-proliferative effect of TEX41 silencing was confirmed by western blot analyses of different proteins involved in the regulation of cell cycle phases. As shown in Fig. 4 C-D a reduction of CDK4 and CDK6 occurred after TEX41 silencing, although in a not significant matter. Moreover, a significant reduction of p27 and an increase of p21 protein levels were detected. These results are in agreement with the growth arrest in the G2-M phase and with the strong activation of the tumor suppressor p53, the most important regulator of cell fate [25].

## Discussion

Different studies suggest that specific lncRNAs could be involved in leukemia and used as biomarkers in the diagnosis and/or prognosis for leukemia patients[17, 20, 26, 27]. Despite the advancement in biological research, little is known about the functional impact of lncRNAs in pediatric B-ALL etiology, progression, and treatment response. The de-regulation of lncRNA has been associated with the carcinogenesis of different human solid tumors such as prostate cancer or hepatocellular carcinoma[28] [12].

Importantly, these molecules are detectable in the body fluids of cancer patients so they could be easily monitored and used as an efficient method for cancer diagnosis and therapeutic monitoring.

Recently, an RNAseq study conducted by our group allowed us to identify the aberrant expression of specific lncRNA*s* in pediatric B-cell acute lymphoblastic leukemia (B-ALL) [21, 22]. Among them, the most up-regulated lncRNA was TEX41 (ENSG00000226674). Few reports are available in the scientific literature about the role of this lncRNA. Specifically, in head and neck squamous cell carcinoma, the expression level of TEX41 is higher than in normal tissues where, by means of inhibiting the expression of the known tumor suppressor miR-340, it induces the expression of COMMD6. These data suggested that the TEX41-miR-340-COMMD6 network represents a potential biomarker for tumor prevention and therapy of head and neck cancer [29]. The tumor-promoting role of TEX41 was also reported in cervical cancer [30]. TEX41 results upregulated also into portal vein tumor thrombosis which develops from hepatocellular carcinoma cells [31].

Here, we analyzed the expression of TEX41 exploiting a cohort of pediatric leukemia patients obtained from St. Jude Children’s Research Hospital cloud databases (https://www.biorxiv.org/content/10.1101/2020.08.24.264614v1). These analyses clearly show that lncRNA TEX41 is primarily expressed in pediatric B-ALL patients instead of T-ALL and AML affected ones.

Moreover, patient’s classification based on the chromosomal rearrangement of leukemic cells at the time of diagnosis disclosed TEX41 expression significantly higher in MEF2D rearranged B-ALL leukemia patients. Recently, fusion genes involving MEF2D have been identified in the precursor B-cell acute lymphoblastic leukemia[32], suggesting a potential connection between TEX41 and MEF2D [33]. In this context, the genomic analyses performed by Gu et al. unveil that the B-ALL patients with MEF2D rearranged present the HDAC9 activation[34], so it is possible that TEX41 too plays a key role in the epigenetic mechanisms linked to this molecular activation process.

The *in silico* analyses were also confirmed by q-RT-PCR experiments conducted on our study population composed of ten young patients affected by common B-cells acute lymphoid leukemia at diagnosis and after chemotherapy treatment. This number of B-ALL patients seems to be small but it is still a representative sample of the disease since pediatric B-ALL is considered a rare disease (https://rarediseases.info.nih.gov/diseases/9240/childhood-acute-lymphoblastic-leukemia). We found that TEX41 is significantly over-expressed in pediatric B-ALL patients compared with healthy subjects. Interestingly, we also found that the expression level of TEX41 decreases after chemotherapy suggesting that this lncRNA is a potential marker of therapy progression and it could be used to understand if the administered therapy is effective. Indeed, functional experiments performed by our research group using a transient silencing approach, disclosed that TEX41 downregulation was followed by impairment of leukemia cell growth followed by cell cycle arrest in the G2-M phase. Furthermore, the silencing of Tex41 was able to induce the upregulation of p21 and, more interestingly, p53. This last finding is of interest as recent studies on lncRNAs strongly suggest that these molecules can control the expression of cell cycle regulators, including the p53 checkpoint [35]. The precise mechanisms by which lncRNAs could influence the cell cycle progression are not known. However, Kitagawa et al. [36] classified their possible mechanism of action into 4 groups: (i) epigenetic regulation of target gene transcription; (ii) regulators of the transcriptional machinery on the target genes; (iii) post-transcriptional modulators of mRNA translation and/or stability; (iv) protein scaffolds able to able promote protein-protein interactions. The influence of TEX41 on the p53 expression is a critical aspect that needs future considerations, especially in light of recent findings proving that specific lncRNAs play an important role in the regulation of the p53 response [37] [35].

Regarding the limitations of our study, they are related to the small number of patients and the difficulty of enrolling during follow-up. However, given the rarity of the disease, our case series, albeit preliminary, represents a significant sample sufficient to speculate on the role of TEX41 as a prognostic biomarker of B-ALL as well as a putative regulator of the cell cycle by influencing the p53 expression.

## Conclusions

Our results indicate that the lncRNA TEX41 is significantly over-expressed in pediatric B-cell acute lymphoblastic leukemia in comparison with healthy subjects and its expression decreases after chemotherapy. Overall our data identify TEX41 as a new diagnostic/prognostic biomarker of pediatric B-ALL, easily detectable in bone marrow blood and potentially useful in patient management. Finally, the silencing experiments on TEX41 showed that this lncRNA can determine a strong upregulation of p53 and cell cycle arrest in the G2-M phase. In this way, TEX41 can be proposed as a possible novel interactor in p53 biology.

## Supplementary Information

**Additional file 1:**

## Data Availability

Starting information regarding TEX41 over-expression in pediatric B-ALL patients is reported in Bio project cod. PRJNA601326 (more information at reference number 22). Additional experimental data are available upon request to the corresponding author.

## Abbreviations

LncRNA: long non-coding RNA
B-ALL: B-cell acute lymphoblastic leukemia
AML: Acute Myeloid Leukemia
TCGA: The Cancer Genome Atlas
PBMC: Peripheral Blood Mononuclear Cells
2′F-ANA: 2′-deoxy-2′-fluoro-beta-D-arabinonucleic acid
PBS: Phosphate Buffer Saline
FBS: Fetal Bovine Serum
IMDM: Iscove’s Modified Dulbecco’s Media
