# Supplementary material for: The lncRNA TEX41 is upregulated in pediatric B-Cells Acute Lymphoblastic Leukemia and it is necessary for leukemic cell growth
Source: Biomark Res. 2021 Jul 7;9:54. doi: 10.1186/s40364-021-00307-7 (PMC8261931; doi:10.1186/s40364-021-00307-7)
Supplement: Supplementary file 1 — Additional file 1: [file 40364_2021_307_MOESM1_ESM.pptx]

## Slide 1
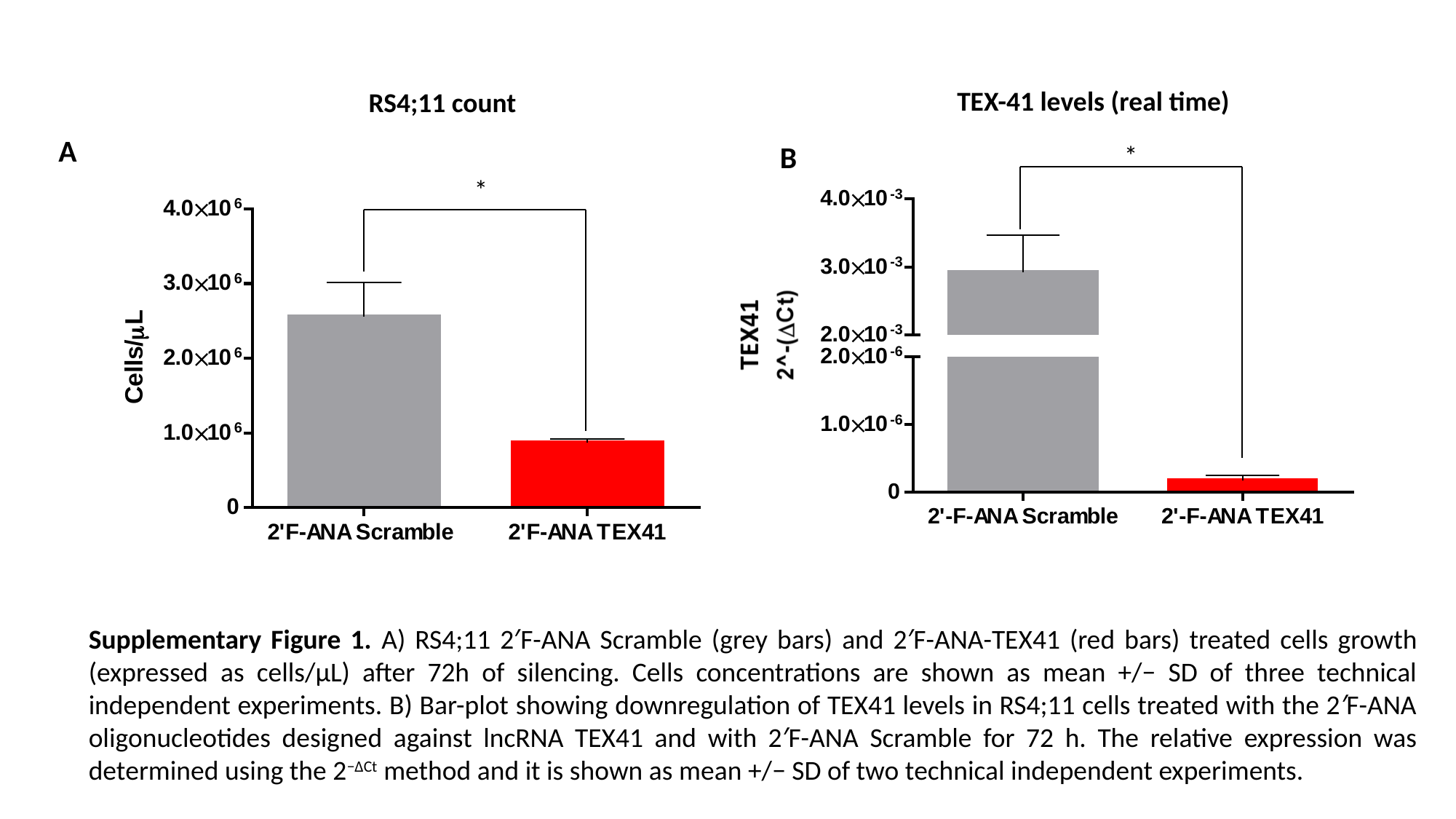

TEX-41 levels (real time)
RS4;11 count
A
B
*
*
Supplementary Figure 1. A) RS4;11 2′F-ANA Scramble (grey bars) and 2′F-ANA-TEX41 (red bars) treated cells growth (expressed as cells/µL) after 72h of silencing. Cells concentrations are shown as mean +/− SD of three technical independent experiments. B) Bar-plot showing downregulation of TEX41 levels in RS4;11 cells treated with the 2′F-ANA oligonucleotides designed against lncRNA TEX41 and with 2′F-ANA Scramble for 72 h. The relative expression was determined using the 2−ΔCt method and it is shown as mean +/− SD of two technical independent experiments.
